# Modulating Polymer Ultrathin Film Crystalline Fraction and Orientation with Nanoscale Curvature

**DOI:** 10.3390/polym15224453

**Published:** 2023-11-18

**Authors:** Roberta Ruffino, Maciej Jankowski, Oleg Konovalov, Francesco Punzo, Nunzio Tuccitto, Giovanni Li-Destri

**Affiliations:** 1 Department of Chemical Sciences and Center for Colloid and Surface Science (CSGI), University of Catania, Viale A. Doria 6, 95125 Catania, Italy; roberta.ruffino@unict.it (R.R.); nunzio.tuccitto@unict.it (N.T.); 2 European Synchrotron Radiation Facility (ESRF), 71 Avenue des Martyrs, CS40220, CEDEX 9, 38043 Grenoble, France; maciej.jankowski@esrf.fr (M.J.); konovalov@esrf.fr (O.K.); 3 Department of Drug and Health Sciences, University of Catania, Viale A. Doria 6, 95125 Catania, Italy; fpunzo@unict.it

**Keywords:** nanostructure, interfacial interactions, nanostructured substrates, strain

## Abstract

We investigated the effect of nanoscale curvature on the structure of thermally equilibrated poly-3-hexylthiophene (P3HT) ultrathin films. The curvature-induced effects were investigated with synchrotron grazing incidence X-ray diffraction (GIXRD) and atomic force microscopy (AFM). Our results demonstrate that nanoscale curvature reduces the polymer crystalline fraction and the crystal length. The first effect is strongest for the lowest curvature and results in a decrease in the out-of-plane thickness of the polymer crystals. On the other hand, the crystal in-plane length decreases with the increase in substrate curvature. Finally, the semi-quantitative analysis of crystal anisotropy shows a marked dependence on the substrate curvature characterized by a minimum at curvatures between 0.00851 nm^−1^ and 0.0140 nm^−1^. The results are discussed in terms of a curvature-dependent polymer fraction, which fills the interstices between neighboring particles and cannot crystallize due to extreme space confinement. This fraction, whose thickness is highest at the lowest curvatures, inhibits the crystal nucleation and the out-of-plane crystal growth. Moreover, because of the adhesion to the curved portion of the substrates, crystals adopt a random orientation. By increasing the substrate curvature, the amorphous fraction is reduced, leading to polymer films with higher crystallinity. Finally, when the thickness of the film exceeds the particle diameter, the curvature no longer affects the crystal orientation, which, similarly to the flat case, is predominantly edge on.

## 1. Introduction

Poly-thiophenes are a class of organic semiconductors that have attracted great interest due to their solubility, easy processability, and environmental stability, which are useful for electronic devices [1,2]. In particular, among the thiophenes, poly-3-hexylthiophene (P3HT) has attracted great interest thanks to its electrical and optical properties [3,4]. It also tends to crystallize, with polymer chains that self-organize into an ordered structure [1,3]. Understanding the crystallization process and controlling the crystals’ dimension are important to improve the performance of devices [3] since a strong correlation occurs between the performance of the device and the solid-state nanostructure [5]. It is known that the crystallization is driven by π-π interactions perpendicular to thiophene rings, leading to anisotropic aggregates forming [6]. Moreover, when the polymer is confined in a thin film, these π-stacking interactions can develop either along the substrate plane or along the direction perpendicular to it. The two preferred lamellar orientations of P3HT thin films are indicated as edge-on orientation, in which the lamellar stacking direction is perpendicular to the substrate surface, while π-stacking interactions are oriented along the substrate plane, and for face-on orientation, the lamellar stacking direction is along the substrate plane [6,7]. In particular, while the edge-on orientation seems to be energetically favored, as it is obtained in close-to-equilibrium conditions, the face-on is a kinetically trapped orientation [7]. Indeed, when the P3HT film is deposited on surfaces, the fast solvent evaporation leads to a kinetically trapped morphology, where the edge-on and face-on orientations coexist. This thermodynamic and kinetic limitation leads to films with low crystallinity. Therefore, a post-deposition process must be performed to enhance the ordered structure [8,9]. Thermal annealing is a common method used to improve the crystalline quality by tuning molecular orientations and enhancing structural order [10]. Indeed, the heat treatment provides the P3HT enough energy to reorganize itself, moving from a structure characterized by a random orientation, in which both the lamellar orientations coexist together with a large amorphous fraction, to an ordered structure where the edge-on orientation is favored in view of its higher thermodynamic stability [3,11,12,13,14,15,16]. 

One of the most effective techniques to record both the P3HT crystal structure and orientation is X-ray diffraction as, in the case of edge-on predominant orientation, the 2D diffraction pattern shows an out-of-plane signal corresponding to the (100) polymer chain folding Bragg peak. This peak is generally accompanied by an additional signal related to the π-stacking, corresponding to (020) Bragg peak, along the substrate plane [3,14]. Moreover, it is essential to consider that the cooling rate also affects the structural order. In particular, it was observed that slow cooling can increase the density of π-stacked ordered structures upon annealing at the polymer melting temperature [17]. Therefore, slow and controlled cooling improves the crystalline quality with strong crystal orientation [18]. Finally, the crystallization of polymer thin films is believed to be initiated by heterogeneous nucleation at the polymer/substrate interface, where the amorphous regions are consumed, resulting in a smooth film characterized by strongly oriented crystallites [14,19]. Therefore, the crystalline structure and orientation are affected by the polymer molecular properties, surface nature, and polymer/substrate interactions [20,21,22]. It is in this perspective that, by preparing nanostructured substrates consisting of monodisperse silica particle monolayers with diameters comparable to the P3HT crystal sizes, we demonstrated that the substrate-induced nanoscale deformation, together with the surface energy balance, drives the crystallization of P3HT ultrathin films [23]. Furthermore, we observed that the substrate curvature already affects the orientation of fast-forming unannealed P3HT lamellae [24]. In the present work, we investigate the role played by the substrate nano-curvature on the crystal nucleation and growth of annealed P3HT films. We will show that the substrate nano-curvature affects not only the crystal orientation and size but also the overall crystalline fraction of the film.

## 2. Materials and Methods

### 2.1. Chemicals

Regio-regular P3HT with Mw: 54,000 and polydispersity 2.3, CHCl_3_, NH_4_OH, and H_2_O_2_ were purchased from Sigma-Aldrich (Milan, Italy) and used as received. Aqueous suspension of silica particles (5% *w*/*v*) with nominal diameters of 50 ± 10, 143 ± 4, 235 ± 10, 304 ± 20, and 403 ± 10 nm was purchased from Microparticles GmbH, Berlin (Germany), and used as received. 

### 2.2. Substrate Preparation

Hydrophilic substrates were obtained by treating silicon wafer (100) with a piranha basic solution (H_2_O, NH_4_OH, and H_2_O_2_, ratio 5:1:1) at 60 °C for 10 min [25].

Nano-curved substrates were obtained by spin-coating the colloidal dispersion of silica particles on piranha-cleaned flat silicon wafers. The spin-coating parameters were adjusted to ensure uniform hexagonally packed arrays with a specific curvature equal to the inverse of particle radii [24]., thus ranging from 0.0049 nm^−1^ to 0.0400 nm^−1^. The so-obtained curved substrates were annealed at 90 °C for 10 min to remove the residual water and then made highly hydrophilic with basic piranha solution treatment. 

A 5 mg/mL P3HT chloroform solution was spin-coated at 4000 rpm for 30 s, leading to films with a thickness of 67.5 ± 6.8 nm, regardless of substrate curvature, as measured by profilometry and UV–Vis characterization [24].

Finally, the polymeric films were thermally annealed in vacuum at 250 °C, P3HT melting temperature [26] for 30 min, then slowly cooled at 3 °C/min to room temperature. 

### 2.3. Morphological Characterization

Morphological characterization was performed using a Nanoscope IIIA-MultiMode atomic force microscope (AFM) Digital Instruments (Santa Barbara, CA, USA) used in tapping mode. Images were recorded using Tap 300 G silicon probes from Budget sensors (Wetzlar, Germany), with a nominal resonance frequency of 300 kHz. The statistical analysis of the P3HT lamella length was obtained with an open-source program coded with MATLAB (R2023b), FiberApp (downloaded via Github) [27]. This program allows for the tracing of the lamellae in AFM images to determine their coordinate and, therefore, the lamellae length distribution.

### 2.4. Structural Characterization

The structural characterization was performed at the ID10 beamline of the European Synchrotron Radiation Facility (ESRF), Grenoble (France), using grazing incidence X-ray diffraction (GIXRD). A 22 keV ± 3.1 eV X-ray beam was employed to record the GIXRD pattern, with an incident beam angle of 0.064 degrees, i.e., 80% of the total reflection at the critical angle. A Pilatus 300 k 2D detector collected the diffracted beam pattern at 389.7 mm from the sample. Samples were placed inside a sample holder covered by a Kapton dome and filled with helium.

A geometrical correction was performed on each diffraction pattern, followed by the conversion from the pixel matrix to scattering vector, q, to extract the 1D profiles by line cutting along the desired directions.

The obtained peaks were then fitted with a Lorentzian equation.

## 3. Results

Reference topographic images of the flat silicon substrate and of the as-deposited particle monolayers are reported in the Supporting Information (Figure S1). The deposition protocol allowed for obtaining hexagonally close-packed silica particle monolayers with micron-scale homogeneity (Figure S2). 

**Figure 1:**
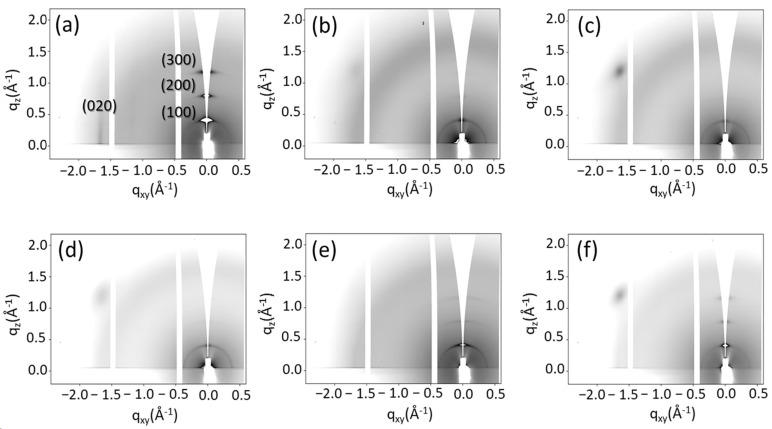
2D GIXRD patterns of P3HT after thermal annealing on flat substrates (**a**) and on substrates having different curvature: 0.00492 nm^−1^ (**b**), 0.00658 nm^−1^ (**c**), 0.00851 nm^−1^ (**d**), 0.0140 nm^−1^ (**e**) and 0.0400 nm^−1^ (**f**). The spot in the left top part of some patterns is a Si reflection, which is recorded in case of non-perfectly homogeneous substrate coverage by particles and polymer.

We have already demonstrated that the spin-coated P3HT thin films homogeneously cover the surfaces without altering the particle monolayer arrangement, and the rapid solvent evaporation leads to the formation of an out-of-equilibrium crystalline structure with low crystallinity [9,24]. In order to increase the crystalline fraction, P3HT films on substrates with different curvature were subjected to thermal annealing and then characterized by synchrotron radiation grazing incidence X-ray diffraction (GIXRD), recording the 2D diffraction patterns in Figure 1 for films on both flat and nano-curved substrates.

The 2D pattern of the flat substrate (Figure 1a) shows the peculiar signature of the edge-on orientation of the P3HT lamellae, with high-intensity signals along the q_z_ direction, corresponding to the out-of-plane lamellar stacking (100) Bragg peak at approximately 0.45 Å^−1^ and the higher-order peaks, namely (200) and (300). Vice versa, the π-stacking generates an in-plane (020) Bragg peak along the q_xy_ direction at approximately 1.70 Å^−1^. After thermal annealing, the crystalline fraction increased, with a predominance of edge-on lamellar orientation, consistent with previous literature reports [28]. 

The 2D patterns recorded on nano-curved substrates (Figure 1b–f) show different behavior, with a ring of uniform intensity at about 0.45 Å^−1^ and a broad halo at 1.70 Å^−1^, corresponding, respectively, to (100) and (020) Bragg peaks. We have already observed similar results for the non-equilibrated films [24]. In that case, the ring was related to the variation in the lamellae orientation with respect to an ideal baseline of the flat substrate. In other words, lamellae adhere to the particle surface with both edge-on and face-on orientations; therefore, the orientation with respect to the sample baseline is random [24]. 

**Figure 2:**
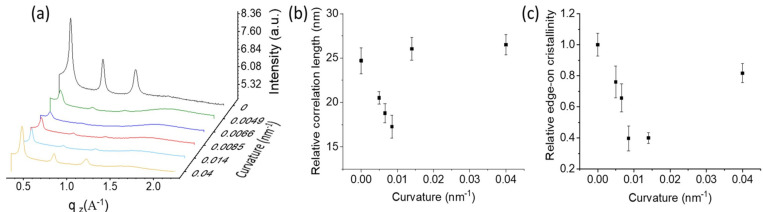
1D profiles were extracted from 2D patterns along the qz direction for different surface curvature (**a**). Semi-quantitative information was obtained by performing a Lorentzian fit of the (100) Bragg peak, and in particular, the relative correlation length (**b**) and the relative edge-on crystallinity (**c**) were determined.

Similarly, thermal annealing favors the edge-on lamellae orientation with respect to the curved portion of the substrates, but to the ideal baseline, the lamellae adopt different orientations, recording, also after the annealing, a narrow ring with uniform intensity. However, with the increase in surface curvature, two more weak signals appear (Figure 1e,f) along the q_z_ direction, corresponding to the higher-order peaks (200) and (300) (Figure 2a), as observed for flat substrates (Figure 1a). 

Semi-quantitative information can be obtained by fitting 1D profiles extracted from the 2D patterns along q_z_ (Figure 2a). The results show that the (100) Bragg peak position is unaffected by the curvature, with a lamellar spacing d_100_ ≈ 14.7 Å, regardless of the substrate curvatures. This proves that the nano-curvature does not affect the P3HT crystalline structure. Similar interplanar distances after thermal annealing have already been reported in the literature [29,30].

From the width at half height (FWHM, w) of the diffraction peak, the out-of-plane relative correlation length (Figure 2b), i.e., the average crystalline size, 2π/w can be determined. However, the experimental apparatus did not account for instrumental broadening as the 170 μm broad detector pixels are unsuitable for high-resolution diffraction measurements. Therefore, the observed peak variation can only be considered as a general indication of the crystalline size variation with curvature and thermal annealing. It should be mentioned that the relative correlation length recorded here on flat substrates is consistent with previously reported P3HT out-of-plane crystal thicknesses [31,32,33]. 

The increase in surface curvature results in an expansion in the full width at half maximum (FWHM) of the diffraction peak (Figure 2b), with a threshold curvature value of 0.0085 nm^−1^. A further increase in curvature leads to a decrease in the peak width.

In order to investigate the orientation distribution of lamellae, we determined the relative variation in the edge-on crystallinity with the curvature from the (100) intensity signal along q_z_. The (100) peak intensity on the flat substrate was used as a normalization factor. The results, reported in Figure 2c, show a decrease in the edge-on crystallinity, enhanced by the substrate nano-curvature up to 0.014 nm^−1^, while the orientational randomization is less marked for the 0.04 nm^−1^ nano-curvature, which shows, similarly to the reference flat substrate, predominant edge-on orientation.

**Figure 3:**
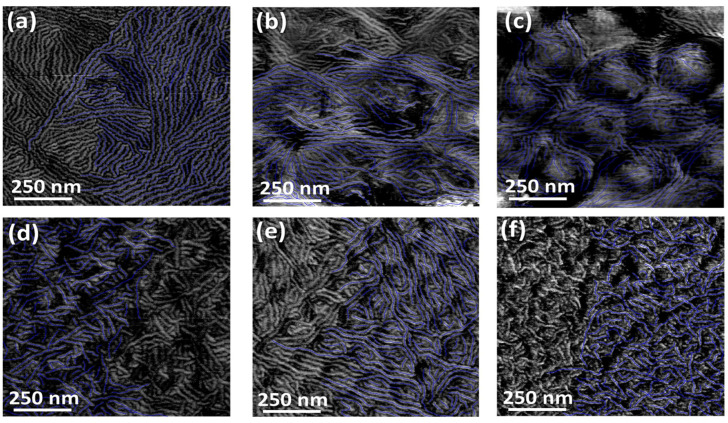
1 × 1 µm^2^ AFM phase images of annealed P3HT films on a flat substrate (**a**) and on substrates having different curvatures: 0.00492 nm^−1^ (**b**), 0.00658 nm^−1^ (**c**), 0.00851 nm^−1^ (**d**), 0.0140 nm^−1^ (**e**) and 0.0400 nm^−1^ (**f**). The blue traces mark the exemplificative lamellar tracing for each image, used to determine the lamellae length distribution.

Further information about polymer crystallization can be obtained from the morphological characterization performed with atomic force microscopy (AFM). The AFM-phase images, reported in Figure 3, show a homogeneous distribution of polymeric lamellae on both curved portions and interstices between particles. However, the substrate curvature significantly influences the length distribution, as determined by tracing the lamellae following the procedure described above. 

**Figure 4:**
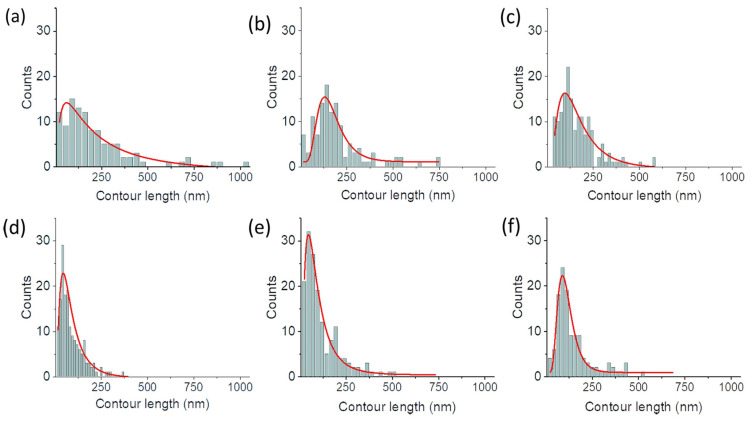
Lamellae length distribution, after annealing, on flat substrate (**a**) and on substrates having different curvatures: 0.00492 nm^−1^ (**b**), 0.00658 nm^−1^ (**c**), 0.00851 nm^−1^ (**d**), 0.0140 nm^−1^ (**e**) and 0.0400 nm^−1^ (**f**), where the contour length is the lengths of the traced lamellae in the AFM phase image, while the count is the number of traced lamellae with that given length. The red lines on different plots represent the log-normal fit of the distributions.

In this case, the average lamellar length decreases as the substrate curvature increases until 0.0085 nm^−1^, beyond which further increases in curvature do not produce significant variations. This is evident from the histogram distribution of the traced length (Figure 4), showing a progressive reduction in the highest lamellar lengths with curvature up to 0.0085 nm^−1^.

**Figure 5:**
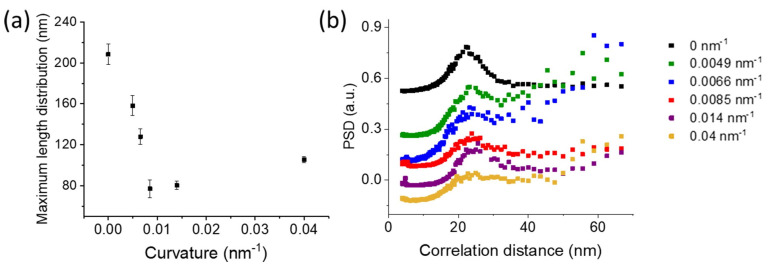
Effect of the surface curvature on the maximum (**a**) of the lamellae length distribution. (**b**) PSD distributions of lamellar thickness of P3HT thin film on substrates having different curvature.

Similarly, the peak of the log-normal fit of the length distribution moves to lower values for curvatures ranging between 0 and 0.00851 nm^−1^, then remains constant (Figure 5a). 

AFM analysis can provide further information on the P3HT crystalline structure by considering the power spectral density (PSD). This tool consists of isotropic Fast Fourier Transform (FFT) filtering, leading to a 1D plot of the reciprocal distance.

Any periodical distance in the AFM images leads to peaks emerging from the sigmoidal PSD plots [34]. In the present case, the periodical signal arises from the constant lamellar thickness generated by the periodic folding of the polymer chain in the crystal [35,36]. Semi-quantitative information was obtained by carrying out a Gaussian fit of the curves (Figure 5b), where the peak position was interpreted as the most probable lamellar thickness. No significant variations in lamellar thickness with curvature were observed, recording mean values of 22.95 ± 0.8 nm. This constant mean lamellar thickness suggests that the uppermost folded polymer chain lays parallel to the substrate plane.

## 4. Discussion

**Figure 6:**
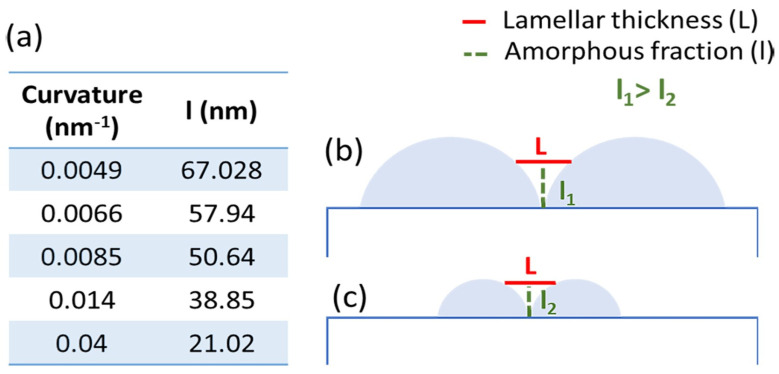
Schematic representation of the nano-curved surface covered by P3HT thin films, where l is the height required to have a distance between adjacent particles at least equal to the folding period L, i.e., the minimum distance between two particles able to accommodate a P3HT lamella. Figures (**b**,**c**) show two different particle dimensions in order to show how l decreases with the reduction of particles diameter leading, as a consequence, to thicker crystallizable film portions above l. (**a**) reported the calculated values of l for all curvatures under investigation.

The above results demonstrate how nanoscale curvature mostly affects the film crystalline fraction and the crystal size. On the contrary, as revealed by the PSD analysis of the AFM images, the crystal arrangement of the uppermost crystals does not change with the substrate curvature. In the assumption that buried P3HT crystals also arrange in an edge-on orientation, i.e., with the lamellar stacking direction perpendicular to the substrate plane and with the folded chain segments parallel to the substrate, the polymer crystallizes only in those substrate portions where the distance between two neighboring particles is equal or higher than the folding period (L = 22.95 ± 0.8 nm). On the other hand, it is reasonable to assume that the bottom polymer film is amorphous. This leads to a reduction in the crystalline fraction as a function of the surface curvature, which agrees with the reduced intensity of the diffraction peaks on nano-curved substrates and the background increase arising from the short-range ordering of the chain segments (see Figure 1). Moreover, the height “l” required to have a distance between adjacent particles at least equal to the folding period (see Figure 6) changes with the particle radius, i.e., with the substrate curvature, as reported in Figure 6a. 

In particular, as the substrate curvature increases, this height diminishes, causing a corresponding decrease in the polymer fraction that occupies the spaces between neighboring particles. This explains why the broad halo at ~1.7 Å^−1^ decreases at higher curvatures. Moreover, the presence of a larger amorphous fraction (see Figure 6a) also explains why the FWHM of the out-of-plane lamellar stacking peak is higher at lower curvatures, i.e., the out-of-plane crystal size is lower. As a significant fraction of the polymer accumulates in the interstices, the ability of lamellae to grow perpendicularly to the substrate plane is reduced. On the contrary, this reorganization favors the in-plane growth, as confirmed by the AFM images (Figure 3), showing that on low-curvature substrates, more extended crystals formed and, among them, the longest ones grew along the interstices between particles. Vice versa, at higher curvatures, the lower amorphous fraction required to fill the interstices between neighboring particles increases the nucleation probability, leading to many short (thicker) crystals, both in the interstices and on the curved portions of the substrate. Finally, when the film thickness exceeds the particle radius for the 0.04 nm^−1^ substrate, the top crystals nucleate and grow on a flat substrate consisting of particles and an interstice-filling polymer film. This leads back to an increase in the relative edge-on orientation (Figure 2c).

Overall, our results demonstrate that the nanoscale curvature decreases both the crystalline length and out-of-plane thickness, consequently increasing the polymer amorphous fraction. This effect, caused by the amorphous interstice-filling fraction, is markedly dependent on the polymer film thickness, as, for a given curvature, the crystallizable polymer fraction above the threshold thickness “l” increases with the film thickness. As a matter of fact, despite the expected stronger distortive effect played by higher curvatures, polymer films deposited on substrates covered with the smallest particles are characterized, because of the lower threshold thickness, by the highest crystalline fraction. Therefore, we expect that our approach can be extended to thicker polymer films, provided that the appropriate substrate curvature is chosen.

## 5. Conclusions

We demonstrated how the geometric strain of substrates can be used to modulate the structure of P3HT thin films. The presence of periodic nanoscale curvature influences the crystalline fraction, morphology, and orientation. In particular, the presence of narrow interstices preventing the chain folding required for crystallization reduces the crystalline fraction, this effect being more relevant at lower curvatures. On the other hand, the morphology and orientation of the crystals are dictated by the residual thickness, i.e., the film fraction lying above the threshold distance between two neighboring particles, enabling chain folding. Low residual thicknesses, i.e., low curvatures, favor in-plane crystal growth, leading to longer lamellae characterized by predominant edge-on orientation. Vice versa, on high-curvature substrates, randomly oriented shorter and thicker lamellae form because of the enhanced nucleation and out-of-plane growth. 

The reported work provides a novel and easy method to modulate the structure of polymer films by exploiting geometric distortion and interfacial interactions with possible effects on the functional properties of the polymer film. Overall, the reported results could pave the way for subtle management of the morphology and structure control of thin films and to a deeper understanding of the self-assembly behavior of confined soft matter by enabling the quantitative determination of fundamental parameters, such as the crystallization enthalpy and its related loss when nanometric strains are applied. This would allow for greater control of the system and the design of advanced devices, in which it is possible to control the individual building blocks and finely modulate the polymeric assembly. In conclusion, our approach might allow for the creation of devices based on the local control of the properties of the system, where the interactions with the substrate are the key parameters for the realization of finely tailored properties.

## Data Availability

The data presented in this study are available on request from the corresponding author.

## Supplementary Materials

The following supporting information can be downloaded at: https://www.mdpi.com/article/10.3390/polym15224453/s1, Figure S1: 1 × 1 µm^2^ AFM height images of flat and nano-curved substrates; Figure S2: 10 × 10 µm^2^ AFM height images of flat and nano-curved substrates.
