# Supplementary material for: Modulating Polymer Ultrathin Film Crystalline Fraction and Orientation with Nanoscale Curvature
Source: Polymers (Basel). 2023 Nov 18;15(22):4453. doi: 10.3390/polym15224453 (PMC10674544; doi:10.3390/polym15224453)
Supplement: Supplementary file 1 [file polymers-15-04453-s001.zip › polymers-2720568-supplementary.pdf]

Supporting Information

Modulating Polymer Ultrathin Film Crystalline Fraction and Orientation with Nanoscale Curvature

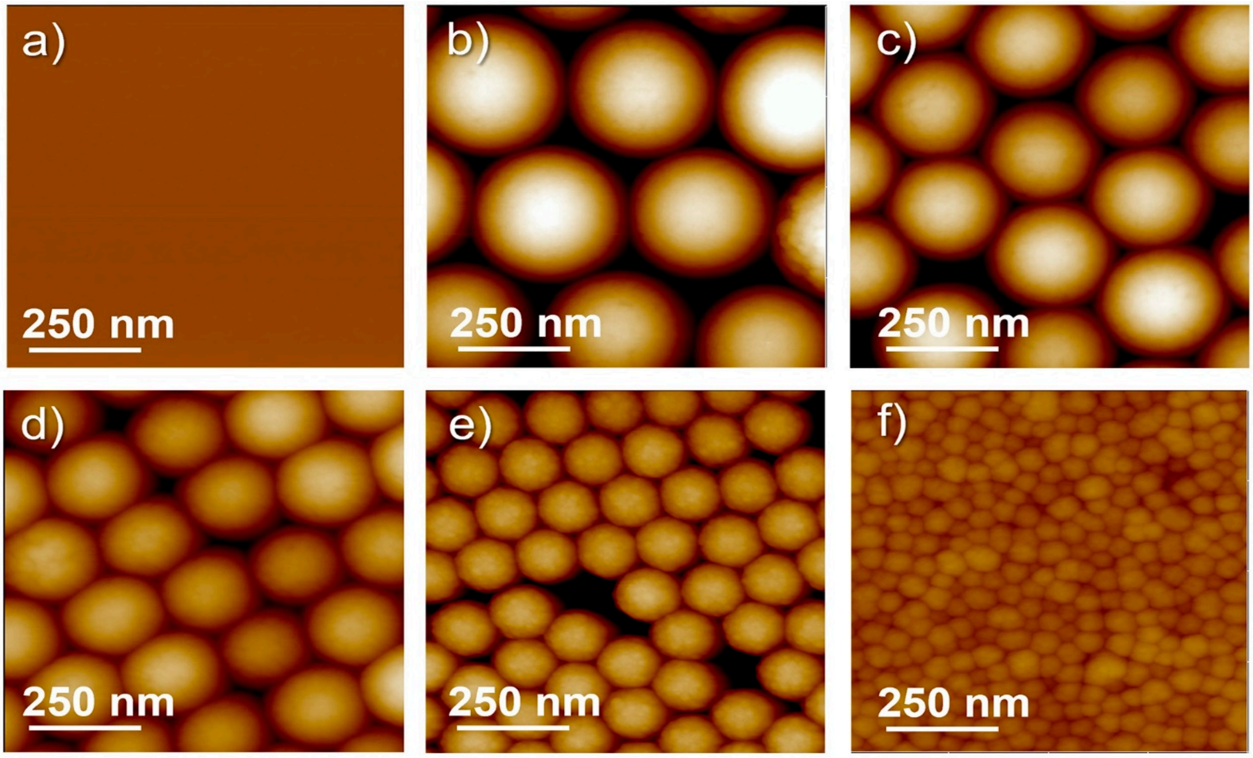

Figure S1. 1 × 1 μm² AFM height images (Z scale 150nm) of bare flat substrate (a) and of bare structured substrates with curvature of 0.00492 nm^{-1} (b), 0.00658 nm^{-1} (c), 0.00851 nm^{-1} (d), 0.0140 nm^{-1} (e) and 0.0400 nm^{-1} (f).

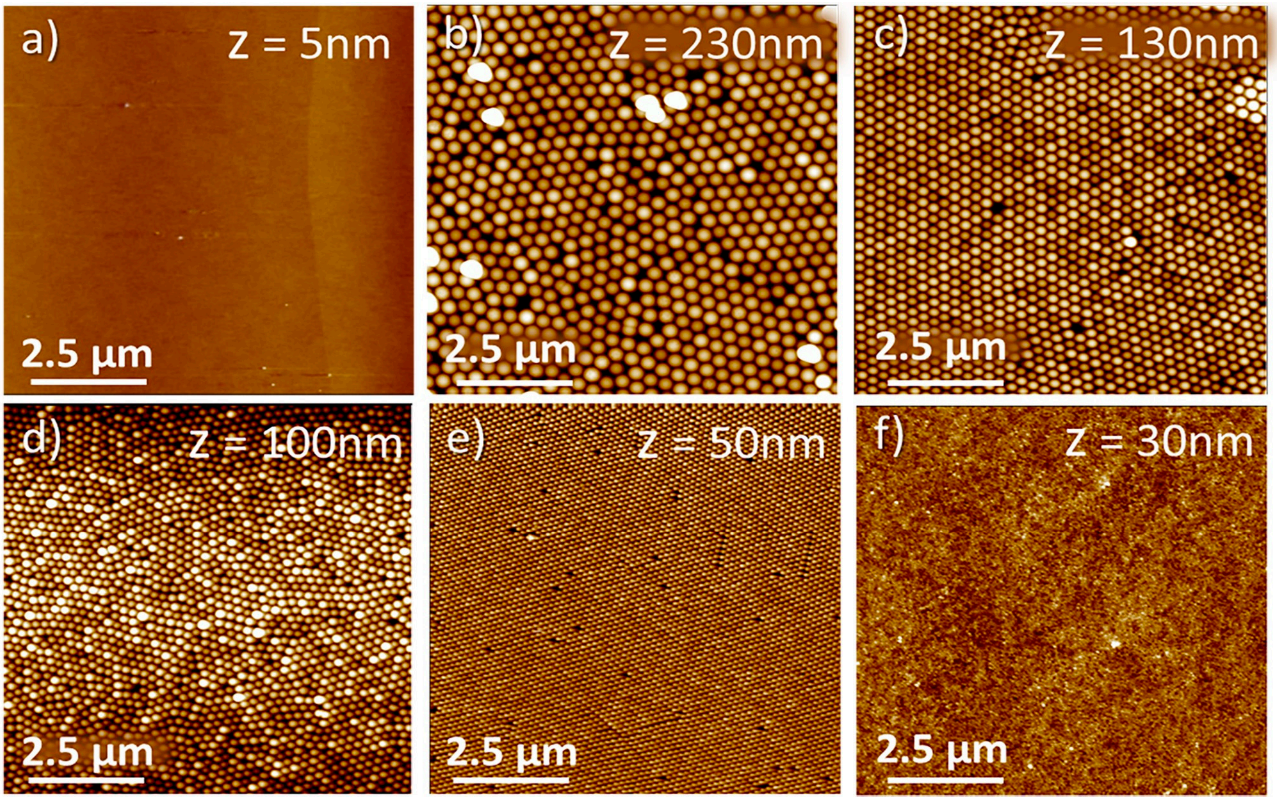

Figure S2. 10 × 10 µm² AFM height images of bare flat substrate (a) and of bare substrates having different curvature: 0.00492 nm^{-1} (b), 0.00658 nm^{-1} (c), 0.00851 nm^{-1} (d), 0.0140 nm^{-1} (e) and 0.0400 nm^{-1} (f).
